# Supplementary material for: Modeling glioblastoma heterogeneity as a dynamic network of cell states
Source: Mol Syst Biol. 2021 Sep 16;17(9):e10105. doi: 10.15252/msb.202010105 (PMC8444284; doi:10.15252/msb.202010105)
Supplement: Supplementary file 5 — Source Data for Figure 3 [file MSB-17-e10105-s001.zip › Figure3A_sourcedata/GSEA_3065/nosigdb_state3.GseaPreranked.1622718426028/LARSSON_STATE_6.html]

Details for gene set LARSSON\_STATE\_6[GSEA]

|  || Dataset | state3 |
| Phenotype | NoPhenotypeAvailable |
| Upregulated in class | na\_neg |
| GeneSet | LARSSON\_STATE\_6 |
| Enrichment Score (ES) | -0.8015675 |
| Normalized Enrichment Score (NES) | -2.8614607 |
| Nominal p-value | 0.0 |
| FDR q-value | 0.0 |
| FWER p-Value | 0.0 |
Table: GSEA Results Summary

  

Fig 1: Enrichment plot: LARSSON\_STATE\_6      
 Profile of the Running ES Score & Positions of GeneSet Members on the Rank Ordered List

  

| PROBE | GENE SYMBOL | GENE\_TITLE | RANK IN GENE LIST | RANK METRIC SCORE | RUNNING ES | CORE ENRICHMENT || 1 | SEPT7 |  |  | 221 | 0.274 | -0.0152 | No |
| 2 | CD74 |  |  | 471 | 0.204 | -0.0363 | No |
| 3 | NEAT1 |  |  | 855 | 0.152 | -0.0748 | No |
| 4 | HNRNPA2B1 |  |  | 863 | 0.151 | -0.0699 | No |
| 5 | TUBB2B |  |  | 904 | 0.148 | -0.0689 | No |
| 6 | CLU |  |  | 988 | 0.139 | -0.0732 | No |
| 7 | TXNIP |  |  | 1317 | 0.114 | -0.1068 | No |
| 8 | DKK3 |  |  | 1538 | 0.101 | -0.1284 | No |
| 9 | CTNNA1 |  |  | 3806 | 0.020 | -0.3896 | No |
| 10 | TUBB2A |  |  | 4022 | 0.015 | -0.4139 | No |
| 11 | HNRNPDL |  |  | 4438 | 0.003 | -0.4618 | No |
| 12 | SOX9 |  |  | 4503 | 0.001 | -0.4691 | No |
| 13 | FOXO3 |  |  | 4960 | -0.010 | -0.5214 | No |
| 14 | SPARCL1 |  |  | 5152 | -0.014 | -0.5430 | No |
| 15 | OLFM2 |  |  | 5382 | -0.020 | -0.5687 | No |
| 16 | MAGED1 |  |  | 5446 | -0.022 | -0.5751 | No |
| 17 | SESN3 |  |  | 5988 | -0.039 | -0.6361 | No |
| 18 | PBX1 |  |  | 6413 | -0.053 | -0.6831 | No |
| 19 | QKI |  |  | 6517 | -0.057 | -0.6929 | No |
| 20 | NFIB |  |  | 6540 | -0.058 | -0.6932 | No |
| 21 | EFTUD2 |  |  | 6558 | -0.059 | -0.6930 | No |
| 22 | FAM20C |  |  | 6618 | -0.062 | -0.6974 | No |
| 23 | COL4A3BP |  |  | 6886 | -0.075 | -0.7255 | No |
| 24 | FYN |  |  | 6924 | -0.077 | -0.7268 | No |
| 25 | HEY1 |  |  | 6933 | -0.077 | -0.7248 | No |
| 26 | C4orf48 |  |  | 6973 | -0.079 | -0.7263 | No |
| 27 | IRS2 |  |  | 7285 | -0.099 | -0.7585 | No |
| 28 | PLEKHB1 |  |  | 7359 | -0.105 | -0.7630 | No |
| 29 | RND2 |  |  | 7413 | -0.109 | -0.7650 | No |
| 30 | CITED1 |  |  | 7470 | -0.114 | -0.7672 | No |
| 31 | GABBR2 |  |  | 7511 | -0.118 | -0.7673 | No |
| 32 | TSC22D4 |  |  | 7610 | -0.129 | -0.7738 | No |
| 33 | TNFRSF21 |  |  | 7822 | -0.155 | -0.7923 | No |
| 34 | FEZ1 |  |  | 7903 | -0.166 | -0.7953 | Yes |
| 35 | SCP2 |  |  | 7919 | -0.170 | -0.7906 | Yes |
| 36 | ID2 |  |  | 7934 | -0.174 | -0.7856 | Yes |
| 37 | ID1 |  |  | 7938 | -0.175 | -0.7794 | Yes |
| 38 | SH3BGRL |  |  | 7984 | -0.182 | -0.7777 | Yes |
| 39 | MXD4 |  |  | 7990 | -0.183 | -0.7713 | Yes |
| 40 | BNIP3L |  |  | 8044 | -0.192 | -0.7702 | Yes |
| 41 | ZMAT3 |  |  | 8086 | -0.201 | -0.7673 | Yes |
| 42 | SOX4 |  |  | 8123 | -0.209 | -0.7636 | Yes |
| 43 | PNRC1 |  |  | 8133 | -0.212 | -0.7566 | Yes |
| 44 | FHL1 |  |  | 8139 | -0.214 | -0.7491 | Yes |
| 45 | CEBPD |  |  | 8158 | -0.217 | -0.7430 | Yes |
| 46 | NLRP1 |  |  | 8171 | -0.220 | -0.7360 | Yes |
| 47 | SKP1 |  |  | 8173 | -0.220 | -0.7278 | Yes |
| 48 | MAP1LC3A |  |  | 8182 | -0.223 | -0.7203 | Yes |
| 49 | HOPX |  |  | 8211 | -0.231 | -0.7148 | Yes |
| 50 | CRYAB |  |  | 8223 | -0.234 | -0.7073 | Yes |
| 51 | FKBP8 |  |  | 8260 | -0.243 | -0.7023 | Yes |
| 52 | MARCH2 |  |  | 8268 | -0.246 | -0.6938 | Yes |
| 53 | ATP6V1G1 |  |  | 8284 | -0.254 | -0.6859 | Yes |
| 54 | MT3 |  |  | 8329 | -0.265 | -0.6810 | Yes |
| 55 | GNAI2 |  |  | 8368 | -0.280 | -0.6748 | Yes |
| 56 | PFDN5 |  |  | 8373 | -0.282 | -0.6646 | Yes |
| 57 | C1QL1 |  |  | 8390 | -0.290 | -0.6555 | Yes |
| 58 | SRP14 |  |  | 8397 | -0.293 | -0.6452 | Yes |
| 59 | MARCKSL1 |  |  | 8415 | -0.300 | -0.6358 | Yes |
| 60 | CTXN1 |  |  | 8418 | -0.301 | -0.6247 | Yes |
| 61 | GABARAPL2 |  |  | 8419 | -0.301 | -0.6133 | Yes |
| 62 | BEX1 |  |  | 8450 | -0.314 | -0.6049 | Yes |
| 63 | ZNF428 |  |  | 8462 | -0.322 | -0.5940 | Yes |
| 64 | SMDT1 |  |  | 8463 | -0.322 | -0.5818 | Yes |
| 65 | LAMTOR4 |  |  | 8464 | -0.322 | -0.5697 | Yes |
| 66 | C4orf3 |  |  | 8475 | -0.327 | -0.5585 | Yes |
| 67 | TUBA1A |  |  | 8499 | -0.341 | -0.5483 | Yes |
| 68 | ID3 |  |  | 8502 | -0.341 | -0.5356 | Yes |
| 69 | PFN2 |  |  | 8503 | -0.342 | -0.5227 | Yes |
| 70 | RPS11 |  |  | 8516 | -0.345 | -0.5111 | Yes |
| 71 | LGALS3 |  |  | 8522 | -0.347 | -0.4985 | Yes |
| 72 | SAT1 |  |  | 8533 | -0.354 | -0.4863 | Yes |
| 73 | FABP7 |  |  | 8536 | -0.354 | -0.4732 | Yes |
| 74 | RTL8C |  |  | 8538 | -0.357 | -0.4598 | Yes |
| 75 | S100B |  |  | 8562 | -0.373 | -0.4484 | Yes |
| 76 | METRN |  |  | 8563 | -0.374 | -0.4343 | Yes |
| 77 | BEX3 |  |  | 8584 | -0.388 | -0.4219 | Yes |
| 78 | ATP6V1F |  |  | 8587 | -0.392 | -0.4073 | Yes |
| 79 | PHPT1 |  |  | 8596 | -0.398 | -0.3932 | Yes |
| 80 | GFAP |  |  | 8610 | -0.413 | -0.3791 | Yes |
| 81 | C1orf122 |  |  | 8622 | -0.429 | -0.3642 | Yes |
| 82 | RPS27 |  |  | 8638 | -0.441 | -0.3492 | Yes |
| 83 | PTMS |  |  | 8642 | -0.447 | -0.3327 | Yes |
| 84 | EEF1A1 |  |  | 8646 | -0.450 | -0.3160 | Yes |
| 85 | TOMM7 |  |  | 8666 | -0.470 | -0.3005 | Yes |
| 86 | SNX3 |  |  | 8668 | -0.473 | -0.2827 | Yes |
| 87 | RPL34 |  |  | 8671 | -0.476 | -0.2649 | Yes |
| 88 | COMMD6 |  |  | 8683 | -0.496 | -0.2475 | Yes |
| 89 | RAB31 |  |  | 8696 | -0.514 | -0.2295 | Yes |
| 90 | FABP5 |  |  | 8699 | -0.518 | -0.2101 | Yes |
| 91 | BRI3 |  |  | 8706 | -0.529 | -0.1908 | Yes |
| 92 | OST4 |  |  | 8722 | -0.575 | -0.1708 | Yes |
| 93 | SERF2 |  |  | 8724 | -0.594 | -0.1485 | Yes |
| 94 | CCNI |  |  | 8731 | -0.624 | -0.1256 | Yes |
| 95 | C9orf16 |  |  | 8734 | -0.642 | -0.1016 | Yes |
| 96 | TPT1 |  |  | 8735 | -0.644 | -0.0773 | Yes |
| 97 | FTH1 |  |  | 8750 | -0.965 | -0.0425 | Yes |
| 98 | FTL |  |  | 8751 | -1.124 | -0.0000 | Yes |
Table: GSEA details [plain text format]

  

Fig 2: LARSSON\_STATE\_6: Random ES distribution      
 Gene set null distribution of ES for **LARSSON\_STATE\_6**
